# Radiation Synovectomy: An Enticing Treatment Option for Inflammatory Joint Pain

**DOI:** 10.1155/prm/8887391

**Published:** 2025-05-13

**Authors:** Ashutosh Dash, Tapas Das

**Affiliations:** ^1^Raja Ramanna Fellow, Bhabha Atomic Research Centre, Mumbai 400085, India; ^2^Radiopharmaceuticals Division, Bhabha Atomic Research Centre, Mumbai 400085, India; ^3^Homi Bhabha National Institute, Anushakti Nagar, Mumbai 400094, India

**Keywords:** radiation synovectomy, radiolabeled colloid, radiolabeled microparticle, rheumatoid arthritis, synovectomy, synovial joints

## Abstract

Radiosynovectomy (RSV) represents an advanced therapeutic modality in nuclear medicine, designed to treat chronic inflammatory joint disorders that are unresponsive to conventional therapies. This targeted approach involves the intra-articular administration of radioactive microparticles containing a β^−^-emitting radionuclide, selectively eradicating the inflamed synovial membrane while preserving surrounding tissues. As a minimally invasive, nonsurgical procedure routinely performed in outpatient settings, RSV offers a compelling alternative to more invasive interventions. Over time, RSV has evolved significantly, transitioning from the empirical use of radiocolloids to the development of specialized agents tailored for different joint types. Advancements in this field continue to explore a variety of β^−^-emitting radionuclides with unique emission characteristics, integrated into novel microparticles to improve both specificity and therapeutic efficacy. The selection of an optimal radionuclide hinges on critical nuclear and chemical properties, ensuring effective binding to microparticles and delivering favorable clinical outcomes. This review examines the evolution of RSV in joint disorder management, detailing its mechanisms of action, key factors influencing radionuclide and microparticle selection, and the methodologies involved in their development and production. Additionally, it provides an overview of commonly used radionuclides and microparticles, evaluating their effectiveness within the ever-evolving landscape of RSV.

## 1. Introduction

Joint disorders resulting from inflammation can lead to joint pain, degeneration of cartilage, accumulation of crystals, or infectious processes. The phenomenon of joint inflammation epitomizes one of the most prevalent health concerns worldwide and constitutes a considerable contributor to disability. The pain associated with joint inflammation may impose functional limitations, thereby impacting the emotional health of affected individuals and reducing the overall quality of life for patients.

Amid the spectrum of joint disorders, rheumatoid arthritis (RA) has emerged as one of the prominent contributors, characterized by an abnormal proliferation of synovial tissue, which affects approximately 1%-2% of the global population [1]. The progression of joint deterioration in RA transpires in stages, commencing with inflammation of the synovial membrane, which leads to pain and swelling, subsequently followed by heightened cellular proliferation and pannus formation, culminating in synovial thickening. Thereafter, the cells of the inflamed membrane secrete enzymes that degrade the surrounding bone and cartilage, culminating in joint deformities, discomfort, and constrained mobility. In the terminal stages of the disease, fibrous tissue may undergo ossification, causing complete immobility of the joint [2]. RA is recognized as the most common autoimmune disorder and one of the most prevalent forms of arthritis [3]. Typically, RA emerges in the twilight of adulthood [4] and potentially leads to complications including the inflammation of the heart and lungs, alongside peripheral neuropathy [5–8]. Considering the extensive systemic ramifications of RA, localized treatment modalities may be preferentially employed to circumvent adverse systemic repercussions.

Hemophilia, a hereditary joint disorder predominantly affecting males, leads to joint complications and functional impairment due to clotting factor deficiencies. Key symptoms include joint hemorrhages and chronic hemophilic synovitis, which, if untreated, can progress to hemophilic arthropathy. Consequently, treatment focuses on effectively managing hemarthroses and preventing recurrent joint bleeding through prophylactic measures [9–14]. A variety of therapeutic modalities, inclusive of systemic treatments such as nonsteroidal anti-inflammatory drugs (NSAIDs), glucocorticoids, and disease-modifying antirheumatic drugs (DMARDs), have been formulated to improve quality of life through pain reduction, prolongation of life expectancy, and sustenance of joint function [15–17]. Localized interventions designed to mitigate joint inflammation and pain have been met with limited success, thereby necessitating the exploration of synovectomy as a means to relieve symptoms and preserve joint health, amid concerns regarding side effects and long-term efficacy [18].

Emerging treatment strategies for joint diseases encompass a range of approaches, including open surgery, arthroscopic synovectomy, and radiation synovectomy (RSV), with RSV demonstrating notable effectiveness in the management of RA and persistent synovial inflammation associated with other conditions. The treatment modality known as synoviorthesis or radiosynovectomy or RSV, first conceived in the year 1924 [19], employs radiopharmaceuticals to administer a β^−^-emitting radionuclide into the joints, aiming to obliterate inflamed tissue, consequently mitigating inflammation, preventing fluid accumulation, and improving joint functionality [20–24].

Over the decades, the deliberate utilization of various radionuclides with distinct radioactive decay properties for managing a wide array of joints, including small peripheral ones, has driven the rapid evolution of RSV. Once a niche and innovative therapy reserved for select patients, RSV has progressively become an essential clinical practice, significantly reshaping the trajectory of joint disease treatment. The therapeutic approach to both acute and chronic inflammatory joint disorders has undergone significant evolution with the introduction and advancement of RSV over the years [25–39].

As RSV continues to evolve, breakthroughs in the field could have profound clinical implications, sparking enthusiasm among researchers and clinicians. This review highlights recent advancements in RSV, providing insights to support researchers and medical professionals working in this dynamic area. We will explore RA, current treatment options, the benefits of RSV, radionuclide selection and carriers, and an assessment of existing radionuclides and their clinical efficacy. Given the interdisciplinary scope of this field, we acknowledge the possibility of unintentional omissions of significant contributions.

## 2. Pathophysiology of Synovial Joints and Synovitis

Before delving into any discussion on RSV, it is pertinent to briefly discuss synovial joints, synovitis, and their pathogenesis, as these concepts form the basis of RSV.

### 2.1. Synovial Joints

Synovial joints possess a synovial cavity containing synovial fluid to facilitate movement between bones. Articular cartilage prevents friction between the bones. A thin synovial membrane lines the inner capsule with synoviocytes. The membrane has collagen and glycosaminoglycan. It contains two mesenchymal layers—the intima and the subintimal layer [40]. Synoviocytes are home to cells adorned with the powers of secretion and phagocytosis. The cellular composition of the intimal layer of the synovial membrane includes two distinct types: Type A cells, primarily responsible for phagocytic and absorptive functions, and Type B cells, specialized in secretion [41–43]. The synovial membrane juggles three cardinal roles: serving as a guardian for solute transportation, purging foreign invaders, and crafting hyaluronan and lubricin [44, 45]. Type B cells stand as sturdy sentinels within joints, ensnaring synovial fluid. Type A synoviocytes play a crucial role in immunity, while Type B synoviocytes regulate nutrient release. Beneath the synovial membrane lies the subsynovial membrane, which houses vital blood vessels—the only vessels present within the joint environment. Within joints, synovial fluid flows seamlessly, providing lubrication and nourishment to cartilage, while harboring essential cells such as chondrocytes and synoviocytes. Inflammatory signals within joints can reduce synovial fluid viscosity while increasing plasma content. The avascular nature of articular cartilage gracefully permits bone choreography, while ligaments elegantly connect bones within joints, providing unwavering support and delicately restricting the range of motion.

### 2.2. Synovitis

On occasion, the synovial tissue might thicken due to congestion and swelling, intruding aggressively into surrounding tissues, resulting in a distressing condition recognized as synovitis. This condition results in thickening of the synovial membrane, which includes blood vessels, immune cells, and cells similar to fibroblasts. Proliferation takes place within the synovial membrane, spreading into the interior compartment of the joint. While the synovium proliferates, the inflamed tissue called pannus migrates from the synovium toward the articular cartilage, ultimately leading to erosions. This can lead to damage to nearby tissues due to reduced blood circulation, culminating in fibrosis and calcification. Therefore, there is a disruption in the normal flow of synovial fluid through the pores of the articular cartilage, resulting in the cartilage becoming thinner, weaker, and less flexible as a result of reduced nutrient diffusion. The invading inflammatory tissues transform into tough fibrous tissue, aggressively occupying the joint space, resulting in erosion of the cartilage and underlying bone, as well as restricting joint movement, ultimately causing discomfort. Synovial fibroblasts contribute to the initiation of inflammation and deterioration of joints by synthesizing inflammatory cytokines and enzymes that break down the extracellular matrix. Synovitis is a condition that triggers the growth of the inner lining of joints, causing damage to cartilage, bone, ligaments, tendons, and ultimately joint destruction. Synovitis is pathologically defined by the development of neovascularization; infiltration of the synovium by various immune cells including lymphocytes, plasma cells, and macrophages; and proliferation of synovial lining cells.

### 2.3. Pathogenesis

While the etiology of RA remains incompletely elucidated, a multitude of distinct pathophysiological processes and molecules unique to RA have been identified and studied in detail. The disease is characterized by synovial hypertrophy and persistent joint inflammation. The proliferation of synoviocytes, coupled with the abnormal activation of various immune-responsive cells including T-lymphocytes, B-lymphocytes, macrophages, neutrophils, dendritic cells, and leukocytes, plays a crucial role in driving inflammation, shaping disease progression, and influencing clinical outcomes. This, in turn, promotes the activation of B cells and macrophages, leading to the release of proinflammatory mediators such as tumor necrosis factor (TNF) and interleukins (ILs). Notably, tumor necrosis factor-alpha (TNF-α), predominantly produced by macrophages, assumes a pivotal role in both the initiation and perpetuation of inflammatory responses, including the induction of IL-1 [46]. An overabundance of TNF-α within the joints fosters the proliferation of synoviocytes and initiates a cascade of mediators that attract inflammatory cells, culminating in joint destruction [47]. Two specific autoantibodies, namely, rheumatoid factor (RF) and antibodies that recognize citrullinated proteins (ACPA), are regarded as significant biomarkers for the diagnosis and prognosis of autoimmune diseases [48].

## 3. Strategies Evolved for the Management of Joint Disorder

Evolution in the management of joint disorders has shifted focus from symptom relief to slowing down structural deterioration. This section delves into the progression of treatment approaches and recent innovations in this realm, aiming to restore joint functionality, enhance the quality of life, impede disease advancement, relieve pain, rectify deformities, and offer preventive measures. The selection of therapeutic measures is contingent on the affected joint, the extent of damage, and the severity of the condition.

In the 19^th^ century, joint ailments were often treated by surgically removing inflamed synovial tissue through procedures like arthrosynovectomy and tenosynovectomy. These methods aimed to clean irregular joint linings with minimal damage, using simple incisions. The first recorded excision of an inflamed synovial membrane occurred in 1877 for tuberculous arthritis, but the method fell out of favor due to the risk of disease relapse [49]. Surgical methodologies evolved over time to improve success rates, incorporating new techniques and prosthetics. The open synovectomy procedure is beneficial for conditions like knee inflammatory arthritis, hemophilic synovitis, pigmented villonodular synovitis, and synovial chondromatosis [50–53]. While surgically removing an inflamed synovial membrane through anterior and posterior incisions proves effective, it entails a prolonged hospital stay, and postoperative complications, and necessitates rehabilitation [53, 54]. The use of surgical debridement techniques has varied over time, particularly with the rise of arthroscopic synovectomy and other noninvasive methods. Today, it plays a more limited role and is generally considered an elective procedure [55]. As an alternative to surgical removal of the inflamed synovial membrane, a less intrusive and effective surgical approach, focusing on removing only the affected areas, arthroscopic synovectomy was first introduced for knee joints in the early 1980s, with Highenboten documenting the initial procedure in 1982 [56]. Although arthroscopy holds an edge over open surgery, it may not be deemed suitable for sizable popliteal masses or involvement beyond the joint [57, 58] and substantiating this perspective is lamentably limited [59].

The use of chemical agents, anti-inflammatory, and radioactive substances for intra-articular injection in the management of inflammatory joint conditions exhibits considerable promise, attributable to its targeted drug release, minimally invasive nature, shorter recovery time, and reduced scarring. Within the realm of chemical synovectomy, the use of osmium tetroxide merits meticulous contemplation [60, 61]. Osmic acid targets inflamed synovial tissue and fat, owing to its pronounced lipid affinity and protein coagulation properties. This leads to necrosis followed by regeneration, thereby reducing pain, stiffness, and swelling, particularly in older male patients with knee arthritis [62–68]. However, the high risk of recurrence at 5 years, coupled with the prospect of chondrocyte necrosis, has considerably dampened the popularity of chemical synovectomy [69–72]. Hyaluronate and corticosteroids have become key agents in medical synovectomy for arthritis management due to their effectiveness and low toxicity. Their intra-articular use has seen a notable surge following Food and Drug Administration (FDA) approval [73, 74]. A wide range of corticosteroid formulations, including triamcinolone acetonide, are utilized in injection regimens [75–91]. However, prolonged use of such agents may result in adverse effects and systemic toxicity, necessitating the need for a careful evaluation of their benefits against potential risks [92–97].

Biologic medications employed in targeted RA therapies encompass TNF-α inhibitors (such as infliximab, adalimumab, etanercept, golimumab, and certolizumab pegol); rituximab, which targets the B cell–specific CD 20 antigen; abatacept, which inhibits T-cell co-stimulation; and tocilizumab, which blocks the IL-6 receptor [98–102]. Biologics are highly effective in managing RA by alleviating symptoms, slowing disease progression, and enhancing quality of life. However, limited comparative trial evidence does not establish a clear preference for one treatment over another. While these therapies modulate the immune system to reduce inflammation, they also increase the risk of infections and potential malignancies due to immune suppression [101, 102].

Researchers are increasingly exploring the use of ionizing radiation for treating joint disorders, focusing on RSV to eliminate diseased synovial membranes while protecting surrounding tissues. This approach involves intra-articular administration of radioactive substances, which has been improved through the use of imaging tools like ultrasound and X-ray for accurate delivery.

## 4. RSV

The journey from the inception and practical usefulness of RSV to its acknowledged status in the management of various joint ailments has been meandering and extensive. Presently, RSV has penetrated deeply into the firmly established therapeutic routines for treating joint conditions. The significant spread and promising potential of RSV are predominantly ascribed to the following.

RSV is typically performed on an outpatient basis and often performs as a day-care procedure [103–109]. It proves to be advantageous for all types of joints, including the smaller ones at the periphery, while maintaining a positive cost-to-benefit ratio [110–113]. It eliminates the necessity for postoperative physical therapy to prevent or alleviate joint stiffness linked to surgical synovectomy. There are no surgical or anesthetic risks involved and no risk of infection due to the potent β^−^ radiation released by the radionuclide in the joint, which eradicates all bacteria. This offers an attractive therapeutic alternative for individuals who are unsuitable candidates for surgical intervention [31]. The extent and length of rehabilitation needed are minimal, with a low dose of effective radiation used in treatment [110]. It allows for the treatment of multiple joints simultaneously on an outpatient basis. Multiple administrations of radioactive doses may be needed to achieve the optimal response, and it is relatively devoid of side effects. Furthermore, the option exists to repeat the process 6 months later in instances of treatment ineffectiveness. In comparison with chemical synovectomy, it is generally more reliable and efficient in deactivating the synovium, leading to improved quality of life and successful management of synovitis. The long-term safety profile of RSV has been well-established, with no reported adverse effects linked to the radioactive substances [109].

### 4.1. Mechanism of Action

Colloids formed within bodily fluids as remnants of tiny organisms, pieces of cells produced when tissues regenerate naturally, and substances absorbed in the intestines, typically removed from circulation through phagocytosis. In this intricate mechanism, colloids in the space outside cells are taken inside by cells like macrophages. While macrophages are commonly located in organs like the liver, spleen, bone marrow, and lymph nodes, they can also be found widely in regions with inflammation.

The commencement of RSV involves the introduction of a radiolabeled particle into either the joint, sheath cavity, or bursa. Right after this application, phagocytosis by Type A synoviocytes enables them to capture the particles. Macrophages, that make up the outer layer of lining cells in the synovial membrane, adhere to particles utilizing a receptor-mediated mechanism and internalize them through an actin-driven process [114].

**Figure 1:**
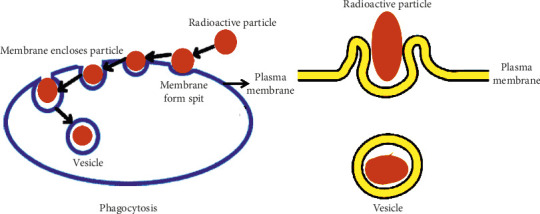
The process of phagocytosis.

The structure of the macrophage membrane alters to envelop the particle within. The phagocytosis process unfolds through the following steps: (1) particles reaching the macrophage membrane surface, (2) particle adhesion to the cell surface, (3) the phagocyte ingesting the attached particle by extending pseudopods around it, and (4) merging of the particle within a phagocytic vacuole known as a phagosome. Numerous factors, such as the dimensions of particles, their morphology, charge on the surface, chemical composition of the surface, and mechanical properties, may influence the phagocytosis mechanism [23, 115–120]. The diagram presented in Figure 1 illustrates the mechanism of phagocytosis.

The β^−^ particles emitted by the radionuclide travel with kinetic energy, ultimately coming to rest near the synovial tissue within a range of few millimeters to 1 cm, depending on the chosen radionuclide. The synchronized progression of coagulation necrosis, fibrosis, and sclerosis in the synovial tissue is observed due to exposure to β^−^ radiation emitted by the radionuclide. The pathologically altered pannus and inflamed synovium undergo destruction through lipid peroxidation, driven by a complex interplay between direct interactions and reactive oxygen radicals. Radioisotopes emerge as more effective in treating diseased synovium, outshining anti-inflammatory or antiproliferative drugs, as the oxygen radicals generated from radiolysis hold the power to induce apoptosis.

Selective irradiation induces histological changes, including a reduction in synovial villi size, increased blood flow, decreased cellular infiltration, and synovial hardening. It is important to note that the radiation does not directly affect cartilage, as it lacks the ability to phagocytose radiolabeled particles and does not contain nerves or blood vessels. Consequently, it cannot perceive pain. The success of the treatment depends on various factors, including joint size, pathological changes in synovial thickness, synovial structure, joint fluid condition, and the degree of inflammation in the synovium [121]. The removal of the swollen synovium not only reduces the flow of synovial fluid but also leads to fibrosis of the synovial membrane and a decrease in inflammatory tissue [122]. The blockage of small blood vessels and capillaries in the synovial membrane can lead to the growth of fibrous tissue, reducing secretory activity and ultimately decreasing pain and stopping joint swelling [123]. This approach prevents additional damage to the joint cavity that would typically result from ongoing immune responses, allowing for the potential for prolonged periods of relief [124].

**Figure 2:**
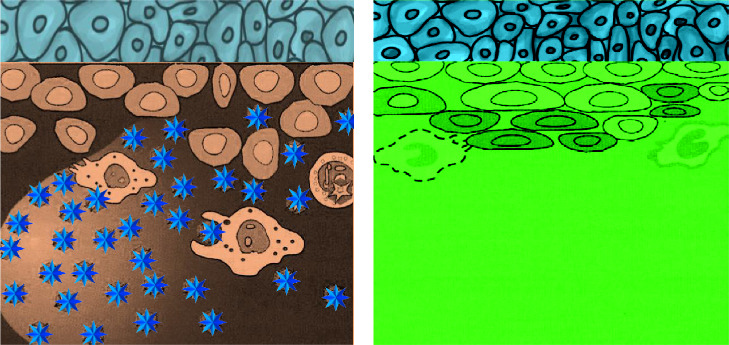
Visual representation of radiosynovectomy. Colloidal particles are prevalent within vacuolar spaces amid the intercellular matrix in synovial biopsies. (a) Blue star particles containing β^−^-emitting radionuclides are engulfed by the enlarged inflamed synovial, where synoviocytes are multiplying, without any impact on the upper layer of cartilage and (b) progressive destruction and hardening of the synovial membrane cells. The radioactive particle must possess a size that is adequately small to facilitate phagocytosis, though not so small as to risk egress from the joint before being phagocytized (the generally accepted size range is typically regarded as being between 2 and 10 μm).

To ensure an even spread of radiolabeled particles in the joint for delivering radiation to the inflamed synovium while minimizing leakage, it is important to use radioactive particles of the right size. Smaller particles provide uniform distribution but may leak beyond the joint, while larger particles resist leakage but may not irradiate the synovial membrane evenly. To avoid the harmful effects of radiation on organs such as the liver, spleen, and lymph nodes, it is important to bind the radionuclide to particles that can be absorbed without being released through lymphatic drainage. The ideal particle size range is between 2 and 10 μm, as this effectively prevents leakage through lymphatic drainage, ensuring optimal performance [125, 126].  Figure 2 illustrates a visual representation of RSV.

The shape of particles is a key element influencing how macrophages take up radiolabeled particles. When particles are being engulfed through phagocytosis, the shape of the particles influences how the macrophage membrane rearranges itself. Research on the internalization of inorganic particles showed that spheres are engulfed more effectively than rods or needles [127]. Other research groups have suggested that the uptake of rod-shaped particles is influenced by their aspect ratio, with extremely high ratios potentially preventing phagocytosis altogether [128]. A noteworthy observation is that hydrophobic targets are significantly more prone to phagocytosis compared to hydrophilic targets [129].

### 4.2. Selection of Radiopharmaceutical

There have been notable advancements in radiopharmaceuticals for RSV due to extensive research conducted in recent years. Radiopharmaceuticals intended for use in RSV are required to meet several specific criteria:

• Radiopharmaceuticals for RSV must contain a β^−^-emitting radionuclide capable of optimal tissue penetration, along with microparticles that deliver precise cytotoxic ionizing radiation to the affected synovial tissue.
• A stable metabolically resistant bond between the radionuclide and microparticles is essential to ensure in vivo stability of the radiopharmaceuticals during RSV.
• The radiopharmaceutical should be able to withstand degradation during storage, transportation, and distribution.
• To effectively target RSV, radiolabeled agents must possess dimensions that allow for ingestion by Type A synoviocytes within the synovial membrane without risking leakage from the joint. The ideal particle size range for these agents falls between 2 and 10 μm [125, 126].
• Preventing the escape of radiolabeled substances from the treated joint is essential due to the potential risk of exposing nontarget organs to harmful radiation. This can be mitigated by using a short half-life radionuclide that decays before leakage can occur.
• The distribution of radiolabeled agents within the joint should be uniform without causing an inflammatory reaction.
• The radiopharmaceutical should be easy to prepare and scalable for large-scale production.
• The ability to formulate radiopharmaceuticals in-house is important for cost-effective production.

### 4.3. Key Steps Involved in Radiopharmaceutical Development

The selection of radiopharmaceuticals for addressing RSV is contingent upon various factors, including the depth of the synovial membrane being targeted, the dimensions of the impacted joint, the dispersion of the radiopharmaceuticals within the joint fluid, and the degree of inflammation. Developing radiolabeled agents for RSV involves several steps that need to be completed before they can be used clinically. These stages involve assessing the size of the joints requiring intervention (large, medium, or small), selecting an appropriate radionuclide based on joint size to avoid cartilage and skin damage, choosing a suitable microparticle for radionuclide attachment, developing a radiolabeling method to ensure optimal efficacy and specificity, and evaluating the quality of radioactive particles before treatment.

### 4.4. Microparticle for RSV

Since it is crucial to keep the radionuclide within the synovial joint to deliver an accurate radiation dose to the synovium, the use of radiocolloids or radiolabeled microparticles, where the radionuclide is bound to a nondiffusible particle, offers a promising and potentially indispensable strategy for RSV.

#### 4.4.1. Selection of Microparticles

The selection of microparticles for RSV is primarily guided by the following key criteria:

• The selected microparticles should be biologically inert, nontoxic, and incapable of eliciting an immune response.
• Biodegradable and biocompatible materials possess the ability to be broken down by natural biological processes within the joint, leading to their elimination from the body without causing harmful effects. These materials are designed to degrade without leaving toxic residues, ensuring rapid clearance without adverse effects on the synovial tissue.
• To ensure effective RSV treatment, microparticles must be capable of remaining suspended in the bloodstream throughout the duration of therapy. It is critical that the density of the microparticle closely mimics the density of human blood, which generally falls within the range of 1.01 to 1.09 g/cm^3^.
• An important characteristic of the microparticles chosen for RSV is their ability to be effectively absorbed by the macrophages located in the synovial lining of the joints. Particularly, macrophages exhibit a preference for hydrophobic particles during uptake.
• The capacity to retain their chemical properties is essential during the process of radiolabeling, which includes various actions such as dividing into small portions, combining, agitating, separating through centrifugation, and subjecting to ultrasonic waves. Additionally, these materials must be able to form physiologically acceptable suspensions or dispersions that remain stable without settling or aggregating. When combined with suitable liquid carriers, such as isotonic saline or phosphate buffer solutions, they should be appropriate for injection or infusion for in vivo administration.
• The selected microparticles for RSV application must possess a stable shelf-life, resistant to aggregation and adherence, and be resilient to various environmental factors such as pH, temperature, and denaturing agents commonly encountered during preparation or storage processes.
• Adequate chemical affinity is necessary to facilitate a secure binding with various radionuclides. The microparticle must establish a chemically stable conjugation capable of withstanding degradation in vivo and sustaining its size within the typical physiological parameters both pre- and post-therapy.
• The microparticles must be resistant to radiological degradation and capable of being easily sterilized.
• The microparticles should be economically viable for commercial production or mass production, with a fast, simple, and consistent preparation process.

#### 4.4.2. Size of the Microparticles

The dimensions of radiocolloidal particles play a significant role in the effectiveness of RSV. The particles need to possess a sufficient size to maintain their integrity within the synovial joint, enabling them to be taken up by the superficial cells of the synovium. Additionally, these particles must have the capability to inhibit excessive leakage from the affected joint. Inadequate particle size could lead to the unwanted leakage of radionuclides toward neighboring lymph nodes and nontarget organs such as the liver and spleen. The key factor to achieve success in RSV lies in choosing the right size microparticle, taking into account various factors elaborated below:

• The dimensions of the microparticles must be sufficiently diminutive to be engulfed by the superficial cells of the synovium, while also avoiding being excessively tiny so as to be swiftly eliminated through biological clearance by diffusion from the joint.
• Although smaller particles allow for an even distribution of radiation on the synovium, they may also lead to a higher occurrence of spread beyond the joint. Conversely, larger particles, while resisting leakage, may result in an uneven distribution that could impact the irradiation of the synovial membrane.
• In order to achieve a consistent radiation dose around the synovium for effective treatment, it is advised to use microparticles with a uniform size distribution. Uneven distribution of these particles could result in unfavorable treatment outcomes—an excess of particles in one area may lead to over-irradiation, while a shortage in another area could result in insufficient irradiation.
• Microparticles should be of a size that allows them to maintain their properties even in the harsh conditions of radiolabeling.
• The microparticles need to stay steady in liquid solutions when subjected to high temperatures for sterilization, as they cannot be effectively sterilized by filtration.
• These particles can have various shapes such as spheres, plates, needles, and rods.
• An optimal particle size of 2 to 10 μm is necessary to avoid leakage from the joint through lymphatic drainage [125, 126].

#### 4.4.3. Microparticles Commonly Used in the Preparation of RSV Agents

A variety of microparticulates are being used to prepare radiopharmaceuticals for RSV, such as glass, ferric hydroxide (FHMA), chromic phosphate, human serum albumin, polylactic acid, Sn colloid, hydroxyapatite (HA), silicate, citrate, chitosan, and nanoaggregates. Although this review does not provide a detailed analysis of each particle, some of the most prominent ones in the field will be briefly highlighted.

##### 4.4.3.1. Glass

Glass is formed through the fusion of metal oxides, creating a remarkable composite material. Through meticulous experimentation, glass spheres of uniform size could be produced. Initially nonradioactive, it undergoes transformation within a platinum crucible at the precise glass-forming temperature. Afterward, the material goes through a process of annealing, crushing, and sieving before being placed in a nuclear reactor to become radioactive. Amid the process of neutron activation, an inactive glass material transforms into radioactive particles of required dimensions. The glass microparticles boast numerous advantages, including exceptional stability, resistance to radiation-induced damage, high insolubility, nontoxicity, compositional versatility, and minimal leaching potential [130, 131]. Despite these splendid qualities, impediments loom large in the form of high density, erratic shape, and stubborn non-biodegradability, hindering the widespread adoption of glass microspheres in RSV. A vast body of literature explores the fabrication of glass microspheres and their applications in RSV [131–133].

##### 4.4.3.2. Chitosan

In the realm of amino-polysaccharides, chitosan stands out with its complex structure composed of 2-deoxy-2-amino-D-glucose units. A positively charged entity arises from the breakdown of chitin, an intricate polymer of β-(1-4)-N-acetyl D-glucosamine. Chitosan's ability to bind radionuclides is attributed to the complex interplay of amino and hydroxyl groups on its molecular structure, resulting in the formation of chelates [134, 135]. Derived through the alchemical process of alkaline hydrolysis of chitin, commonly found within the husks of marine creatures, chitosan emerges as a remarkable compound with a plethora of virtues. It the realm of RSV, it stands apart for its exceptional biocompatibility, lack of toxicity, minimal allergenic potential, and innate biodegradability [136–141].

##### 4.4.3.3. Silicate

Silicate known for its simplicity and scalability offers a wide range of advantages. It is cost-effective, biocompatible, low in toxicity, stable in laboratory and clinical settings, and easily customizable through surface modifications to target-specific radionuclides, making it a versatile material. Precise control over silica particles opens up numerous possibilities, allowing for adjustments in size, porosity, crystallinity, and shape. Furthermore, the surface chemistry of silica particles facilitates the binding of various radioisotopes without the need for specific chelating agents, creating an ideal platform for RSV. The successful use of silicate macroaggregates carrying radionuclides in RSV therapy has made a significant impact across different joints [142–147].

##### 4.4.3.4. Citrate

Citrate is a versatile, nontoxic, easily accessible, and cost-effective compound. The effective chelation properties of citrate in binding metal ions have been utilized in the development of RSV agents [148–151].

##### 4.4.3.5. Sulfide

The intriguing chemical properties and potential biomedical applications of metal sulfide aggregate particles have garnered significant interest. In Europe, ^186^Re-sulfide has received approval for the treatment of various refractory painful arthropathies, though literature on the subject remains limited [149, 152–154].

##### 4.4.3.6. Polylactic Acid (PLA)

Substantial focus has been directed toward the application of PLA owing to its appealing chemical, physical, and biological properties. The utilization of PLA and its derivatives has attracted attention due to their unique mechanical and biological attributes, such as biocompatibility, biodegradability, lack of allergenicity, and nontoxic nature. PLA has been approved by the US FDA for use in the food and pharmaceutical industries since 1970s. PLA is commonly manufactured through various techniques like polycondensation, polymerization, and azeotropic dehydration condensation reaction [155–161]. Classified as an aliphatic polyester, PLA can be tailored with a chelator to efficiently bind radionuclides with a high level of binding stability. PLA microspheres have been employed for immobilizing various radionuclides in RSV [162–164].

##### 4.4.3.7. Hydroxyapatite (HA) [Ca_10_ (PO_4_)_6_ (OH)_2_]

In recent years, there has been a growing interest in utilizing HA microparticles for RSV, owing to their unique physicochemical properties, including biocompatibility, bioresorbability, nonallergenic, nontoxic, and nonimmunogenic nature. Additionally, their remarkable mechanical strength and close resemblance to the carbonated apatite found in human bones and teeth further enhance their appeal. HA is a natural mineral component of the human bone matrix, transforming into Ca^2+^ and PO_4_^3-^ ions through natural metabolic processes which are eventually eliminated from the body within 6 weeks. The surfaces of HA particles harbor reactive sites that facilitate the absorption or covalent bonding with radionuclides to create complexes. Various methodologies such as the sol-gel method, hydrothermal technique, multiple emulsion technique, biomimetic deposition, and electrodeposition have been utilized for the synthesis of HA particles, each offering unique advantages and disadvantages [165, 166]. The remarkable potential displayed by HA particles as a delivery system in RSV has sparked a significant amount of interesting research and innovative therapeutic strategies [167–186]. The remarkable efficacy demonstrated by HA as a carrier molecule for RSV radiopharmaceuticals highlights its crucial role in the research and advancement of RSV therapies.

### 4.5. Preparation of Radiolabeled Particles for RSV

The labeling of microparticles intended for use in RSV can be conducted either during the preparation of the microparticles or afterward. When choosing the labeling method, key considerations should include the simplicity and effectiveness of the labeling process, as well as the stability of the radionuclide particles in vitro and in vivo.

#### 4.5.1. Radiolabeling During the Particle Preparation

The process primarily involves two sequential stages: Firstly, the conversion of radioactive precursors into a uniformly distributed precipitate of a specific inorganic compound through precise experimental conditions, and secondly, the transformation of this precipitate into uniform, small particles. The temperature of the reaction and the pH of the reaction mixture are essential factors in determining the particle size distribution.

#### 4.5.2. Radiolabeling Particles After Their Preparation

In this approach, two methodologies are employed.

##### 4.5.2.1. Neutron Activation of Preformed Particles

In this approach, preformed particles containing the nonradioactive precursor of the radionuclide undergo neutron activation in a nuclear reactor. During neutron activation, the nonradioactive precursor absorbs neutron, converting it into a radioactive nuclide. The benefits of this method encompass avoiding direct contact with radioactivity for radiolabeling, regulating activity levels by managing irradiation time and neutron flux, along straightforward postirradiation processing. The drawbacks of this technique include the requirement of a nuclear reactor for neutron activation, the need for a relatively high neutron activation cross-section to achieve adequate radioactivity, and the susceptibility of particles to radiolytic degradation. Glass has been identified as the most stable matrix for making radioactive particles utilizing this method.

##### 4.5.2.2. Reaction of Radionuclide With Particles

In this approach, nonradioactive microparticles are first prepared and then subjected to radiolabeling with an appropriate therapeutic radionuclide. In contrast to neutron activation of preformed particles, radiolabeling of particles is fundamentally more straightforward. The advantage of this procedure lies in minimizing radiochemical instability resulting from particle irradiation and circumventing logistical challenges linked to handling radiolabeled particles as well as decay loss. Developing a radiolabeling protocol involves leveraging the chemical attributes of the microparticles and radionuclides. It is imperative to determine the precise amounts of each component required during radiolabeling, as excessively high or low concentrations of any component could jeopardize the integrity of the radiolabeled particles.

Diverse techniques utilized for radiolabeling encompass isotope exchange reactions, incorporating a foreign label, covalently bonding, or chelating (forming complexes) the radionuclide with the particles, adsorption, and more. Despite many radiolabeled particles becoming essential radiopharmaceuticals for various clinical uses, their chemistry remains inadequately understood. Further endeavors are necessary to comprehend the chemistry and mechanisms of radionuclide binding. Such insights would facilitate refining particle size and shape, enabling more precise applications in the days ahead.

### 4.6. Radionuclide for RSV

A wide range of radioactive elements is available for use in RSV, and the selection is largely dictated by the size of the specific joint undergoing treatment to ensure optimal therapeutic effectiveness. The criteria for selecting a radioactive element in RSV for a particular joint primarily hinge on its nuclear decay properties, chemical behavior, and logistical aspects of production. This choice of selecting the appropriate radionuclide for RSV is influenced by several key factors as outlined below:

• Radionuclides that emit β^−^ particles, undergo electron capture (EC) and internal conversion (IC) processes, releasing Auger and Coster–Kronig (C-K) electrons, are selected for their precision in delivering targeted radiation to the proliferating layer of the inflamed synovium while safeguarding critical structures like cartilage, bone marrow, and skin from damage.
• The choice of radionuclide is largely dictated by the size of the synovial membrane in the targeted joint. The radiation's penetration depth must align with the thickness of the inflamed synovium, ensuring effective treatment while preventing excessive exposure that could harm the articular cartilage, subchondral bone, or overlying skin. Insufficient penetration can lead to ineffective treatment outcomes, while excessive depths may endanger the cartilage surface.
• High-energy β^−^-emitters, such as ^90^Y and ^188^Re, are frequently utilized for RSV of knee joints. Similarly, medium-energy β^−^-emitters, including ^153^Sm, ^177^Lu, and ^186^Re, are employed in RSV procedures for medium-sized joints such as the glenohumeral joint, elbow, radiocarpal joint, and tibiotarsal joint. In contrast, low-energy β^−^ emitters like ^169^Er and radionuclides emitting Auger and C-K electrons, such as ^117m^Sn, are often advised for smaller joints like the metacarpophalangeal (MCP), proximal interphalangeal (PIP), and metatarsophalangeal (MTP) joints found in the fingers and toes. Additionally, joints such as the distal interphalangeal (DIP), tarsometatarsal (TMT), proximal tibiofibular, and the first carpometacarpal (CMC I) joint of the thumb may also see therapeutic benefits from the use of ^169^Er in RSV treatments.
• The radionuclide should have a half-life long enough to allow practical storage and transportation while ensuring a sustained therapeutic effect. However, it must also be short enough to minimize unnecessary radiation exposure. Additionally, it is crucial to ensure high radionuclidic purity for effective and safe application.
• The radionuclide's therapeutic efficacy is primarily derived from its β^−^ or electron emission. Additionally, a low-abundance γ-emission in the range of 100 to 200 keV is beneficial for precise dosimetric calculations, monitoring radiopharmaceutical distribution within the joint, assessing potential extra-articular leakage, and enabling therapeutic tracking using an Anger gamma camera or single photon emission computed tomography (SPECT) imaging.
• The radionuclide must possess chemical characteristics that enable it to be bound to a variety of particles with diverse chemical attributes, or capable of forming colloidal particles of specified size and quality.
• The radionuclide should be produced using efficient and economical methods to facilitate large-scale availability. Even if a radionuclide possesses ideal therapeutic properties, its widespread adoption in RSV may be hindered if a cost-effective production process is not available.

#### 4.6.1. Gold-198

Gold-198 undergoes decay to ^198^Hg with a half-life of 2.7 days through emission of β^−^ particles, with maximum β^−^ energy of 0.96 MeV, and γ energy of 412 keV accounting for 95.6% of emissions. About 99% of the β^−^ particles have an energy between 0 and 0.96 MeV, with the highest energy range observed in soft tissues extending from 3 to 4 mm. Additionally, approximately 95% of the γ emission occurs within the energy range of 0–412 MeV.

The initial studies on the utilization of ^198^Au in the management of arthritis were first documented in 1963, with the emphasis on managing long-term knee joint swelling by administering 370 MBq (10 mCi) of ^198^Au colloids for treatment [187]. Subsequent to these findings, a multitude of studies have been released in academic journals exploring the use of ^198^Au [188–198].

The benefits and drawbacks of using ^198^Au for RSV are worth considering. The appeal of utilizing ^198^Au lies in its β^−^ energy (0.96 MeV) and tissue infiltration distance of 3–4 mm, making it appropriate for treating large or medium inflamed joints through RSV. Moreover, the half-life of ^198^Au, which is 2.7 days, is seen as sufficient for tasks such as radiolabeling, quality control (QC), and providing treatment to patients. To obtain ^198^Au with high specific activity and in the desired quantity, a metallic natural gold target (natural gold consists 100% ^197^Au) can be irradiated in a reactor and subsequently subjected to straightforward radiochemical processing.

Nevertheless, despite its positive attributes, ^198^Au is associated with significant drawbacks. The β^−^-particle emissions only have a range of 3-4 mm in tissues, which could be insufficient for completely ablating thick synovium in chronic knee effusions exceeding 1 cm in thickness. The release of particles, combined with the relatively long half-life of 2.7 days, results in significant radiation exposure to organs such as the lymph nodes, liver, and kidneys. Additionally, the substantial presence of a γ-ray component (412 keV) in ^198^Au creates a radiation hazard during the preparation of synovial agents, and also contributes to an excessive radiation dose burden on nearby lymph nodes.

#### 4.6.2. Dysprosium-165

Dysprosium-165-FHMA (FHMA macroaggregate) was the initial radiopharmaceutical agent that sparked enthusiasm for the broader implementation of this technology in treating refractory synovitis as a substitute for surgical methods [199–215].

Dysprosium-165 (^165^Dy) has a half-life of 2.33 h and transforms into holmium-165 through the emission of β^−^ particles. The β^−^ particles possess maximum energies of 1286 keV (83%) and 1191 keV (15%), along with a minor γ component with an energy level of 94 keV (3.60%). The γ component can be utilized for monitoring potential leakage of the agent being used for treatment from the targeted site. Furthermore, ^165^Dy has a tissue penetration distance of 5.7 mm.

Like all radionuclides, ^165^Dy has both advantages and disadvantages. One of the positive aspects of ^165^Dy in the context of RSV is its suitability for emitting 1.3 MeV β^−^ particles that can penetrate tissues up to 5.7 mm deep. It also possesses favorable chemical properties for labeling particles; emits γ radiation at 94 keV with an abundance of 3.6%, allowing simultaneous imaging and dosimetry studies; and has a straightforward production process and radiochemical processing for target dissolution.

The short half-life of ^165^Dy plays a crucial role in reducing the consequences of any radioactive discharge from the treated joint. Through the neutron irradiation of enriched ^164^Dy (natural abundance 28%) targets for short times, one can produce ^165^Dy with high specific activity, thanks to the substantial neutron absorption cross-section of ^164^Dy (2840 b). The release of β^−^ particles with moderate energy and γ photons with low energy leads to relatively minor radiation exposure and minimal concerns regarding radioprotection.

Despite these favorable characteristics, ^165^Dy has not been widely accepted, and its usage has not received approval by US FDA because of concerns about possible capsule leaks causing high radiation exposure to organs like the lymph nodes, liver, and kidneys. Furthermore, the short half-life of ^165^Dy requires a nearby nuclear reactor for consistent radionuclide production and necessitates the administration of radiopharmaceuticals soon after preparation.

#### 4.6.3. Erbium-169

Erbium-169 decays with a physical half-life of 9.4 days, emitting β^−^ particles with maximum energies of 342 keV (45%) and 351 keV (55%), as well as γ rays of 110.5 keV (0.0014%). The decay process results in the formation of stable ^169^Tm. Beta particles emitted from ^169^Er can travel up to 1 mm in soft tissue, with an average range of 0.2 to 0.3 mm. Since only a small amount of γ rays are emitted during decay, post-therapeutic scintigraphy is not effective for analyzing distribution. Erbium-169 can be produced through neutron activation of either natural Er (^168^Er has 26.8% natural abundance) or enriched Er (in ^168^Er) target in a nuclear reactor. However, since the thermal neutron capture cross-section of ^168^Er is relatively low (σ = 2.74 b), irradiation of enriched ^168^Er (in ^68^Er) is necessary for efficient ^169^Er production for RSV applications. Radiochemical processing involves the simple dissolution of the irradiated target in an acidic medium with gentle warming, allowing for efficient recovery of the desired radionuclide.

According to several reports, ^169^Er has become the favored choice for RSV of digital joints such as MCP, PIP, and MTP [216–225]. Other joints that can be treated with ^169^Er are DIP, TMT, the proximal tibiofibular joint, and CMC I [30]. [^169^Er]Er-citrate or phosphate colloid has been evaluated in clinical trials for treating RA and osteoarthritis affecting small joints. Clinical studies have demonstrated its effectiveness in reducing pain, swelling, and inflammation in patients with chronic arthritis [220, 221]. Since ^169^Er has minimal γ emission, attempts have also been made to investigate hybrid imaging agents or dual-labeled tracers to improve post-therapy monitoring [225].

The dosage and quantity of ^169^Er activity given usually vary based on the particular dimensions of the joint that needs to be treated [225]. The recommended doses for different joints are as follows: 10–20 MBq for fingers, 20–40 MBq for wrist joints, and 20–80 MBq for thumbs. It is possible to apply the same mixture to multiple joints in a single session; however, the total dose of ^169^Er administered must not exceed 750 MBq per session.

The utilization of ^169^Er in RSV comes with both advantages and disadvantages. The key advantages of using ^169^Er is its suitable β^−^ energy for treating digital joints, the feasibility of reactor-based production, and its 9.4-day half-life, which allows for efficient transportation of radiopharmaceuticals to users.

Despite these advantages, ^169^Er also has drawbacks, including high production costs due to the moderate neutron activation cross-section of ^168^Er and challenges in capturing post-therapeutic scintigraphy images because of its low γ-ray emission.

#### 4.6.4. Phosphorus-32

Phosphorus-32 transforms into sulfur-32 through the emission of β^−^ particles with an energy of 1.71 MeV and a half-life of 14.3 days. The average depth of tissue penetration of β^−^ particles emitted during the decay of ^32^P is 2.2 mm, with the deepest point reaching 7.9 mm. The production of ^32^P in a nuclear reactor occurs through two distinct methods both extensively discussed in existing literature [226]. In brief, ^32^P is primarily produced on a large scale using the ^32^S(n,p)^32^P reaction, which yields ^32^P in a no-carrier-added (NCA) form. However, one of the major drawbacks of this method is its poor production yield. Alternatively, ^32^P can also be produced through the thermal neutron activation of natural elemental phosphorus (^31^P, 100% naturally abundant). Although this method results in ^32^P with low specific activity, it offers a simpler and more convenient approach for producing ^32^P, suitable for RSV application.

The radioactive decay properties of ^32^P make it particularly suitable for RSV procedures on knee joints, with recommended activity levels ranging between 37 and 54 MBq [227]. Numerous studies have investigated the application of ^32^P for RSV of knee joints, as evident from the extensive body of literature [227–239].

The use of ^32^P in RSV requires a careful assessment of its pros and cons, similar to any radionuclide. The positive aspects of ^32^P encompass its cost-effective availability in NCA form, the appropriateness of its 1.71 MeV β^−^ emissions for treating inflamed large joints like the knee, its favorable chemical properties for radiolabeling, and the logistical benefits of easy shipment to locations far from the reactor production site due to its relatively extended physical half-life.

On the flip side, the hurdles linked with ^32^P involve challenges such as the complexity of obtaining post-therapeutic scintigraphy images for dosimetric analysis because of the absence of γ rays suitable for monitoring, the risk of receiving additional bone dose from potential activity leakage from the joint, and the susceptibility to damage in articular cartilage and growth plates caused by the high-energy β^−^ radiation [239].

#### 4.6.5. Holmium-166

Holmium-166 undergoes radioactive decay to produce stable Erbium-166 with a maximum β^−^ energy of 1.85 MeV (50.0%) and 1.77 MeV (48.7%), having a half-life of 26.83 h. Additionally, it releases 81 keV γ photons (6.7%), which can be utilized for external imaging purposes. The extent to which β^−^ particles penetrate the soft tissue is approximately 8.7 mm. ^166^Ho has an energy deposition profile where most of its energy is concentrated in a small 2.1-mm-diameter area, with the rest of the energy spread out in a wider area ranging from 2.1 to 8.7 mm in diameter [240].

Holmium-166 is produced through the direct activation of natural holmium by neutrons. Natural holmium consists solely of ^165^Ho, and exists in 100% natural abundance. The thermal activation cross-section of ^165^Ho measures 64.7 b, making it conducive for the production of substantial quantities of ^166^Ho with a relatively high specific activity by simple neutron irradiation. This method is cost-effective and feasible through the utilization of medium flux research reactors. The potential of ^166^Ho in RSV has sparked significant interest and prompted various innovative research endeavors [163, 164, 240–246].

The primary benefits of utilizing ^166^Ho include its moderate half-life, which reduces the hazards linked to potential leakage of the radionuclide from the joint cavity, sufficient range of β^−^-particles for synovial ablation, cost-effective accessibility, and the potential for simultaneous diagnostic monitoring due to the presence of 81 keV γ photons. However, the logistical difficulty of transporting ^166^Ho-based RSV agents due to its physical half-life of 26.83 h could hinder their distribution to nuclear medicine centers far from reactors and restrict their overall use.

#### 4.6.6. Samarium-153

Samarium-153 decays into ^153^Eu by emitting β^−^ particles, which has a physical half-life of 46.27 h (1.93 days). The β^−^ particles released from the decay of ^153^Sm have energies of 0.81 MeV (20%), 0.71 MeV (49%), and 0.64 MeV (30%). These particles have average and maximum penetration ranges of 0.8 mm and 3.1 mm in soft tissues, respectively. Additionally, ^153^Sm emits 103 keV γ-photons with an abundance of 28%, facilitating imaging using conventional γ-ray cameras. The range of penetration demonstrated by ^153^Sm is deemed sufficient for synovial ablation while ensuring the preservation of adjacent anatomical structures outside the joint, such as cartilage or bone, from damage. The depth of penetration of β^−^ particles originating from ^153^Sm is not only conducive for performing synovial cauterization on joints of moderate size like the hip, shoulder, elbow, wrist, ankle, and subtalar joint, but also holds the potential to enhance the radionecrosis effect through the utilization of increased radioactivity levels.

Production of ^153^Sm involves neutron irradiation of enriched ^152^Sm targets in a nuclear reactor. This process triggers the ^152^Sm(n,γ)^153^Sm reaction, which has thermal and resonance neutron cross-sections of 210 and 3020 b, respectively. ^153^Sm can be economically produced with high specific activity due to its significant thermal neutron capture cross-section. Several research studies utilizing ^153^Sm in RSV have been documented in the contemporary literature [138, 168–170, 180, 247–251].

The key advantages of using Samarium-153 (^153^Sm) include its optimal half-life, high thermal neutron capture cross-section for efficient production, and 103 keV γ emission for diagnostic imaging. Although ^153^Sm has a β^−^ energy approximately 2.8 times lower than ^90^Y, this limitation can be compensated by administering a higher dose of ^153^Sm. This approach ensures an adequate therapeutic effect on the synovial tissue, a strategy supported by its high thermal neutron capture cross-section, which enables efficient production. The comparatively shorter half-life of ^153^Sm (46.27 h) reduces the radiation risks associated with potential leakage of the radioisotope from the synovial cavity, ensuring safer application in therapeutic procedures.

While there is a certain appeal in using ^153^Sm for RSV of medium-sized joints, it is not devoid of downsides and restrictions. The physical half-life of ^153^Sm presents a logistical challenge for transporting radiopharmaceuticals to distant locations, and the need for an enriched ^152^Sm target to produce ^153^Sm renders it less cost-effective compared to ^166^Ho. The relatively high γ abundance of around 28% poses a risk of collateral damage to surrounding healthy tissues.

#### 4.6.7. Rhenium-186

Rhenium-186 decays into Osmium-186 (^186^Os) by emitting β^−^ particles, with a physical half-life of 90 h. These β^−^ particles carry a maximum energy of 1.08 MeV and have a mean tissue penetration depth of 0.92 mm. Moreover, ^186^Re releases γ photons at 137 keV (9.42%), which are ideal for scintigraphic imaging postprocedure. Its limited soft tissue penetration confines radiation exposure to structures near the joint cavity. The moderate energy β^−^ particles emitted by ^186^Re make it suitable for RSV of medium-sized joints like the hip, shoulder, elbow, wrist, ankle, and subtalar joint. The 3.8 d half-life of ^186^Re allows ample time for preparation, quality assurance (QA), and distribution of radiopharmaceuticals worldwide. The production of ^186^Re in nuclear reactors worldwide via the ^185^Re(n,γ)^186^Re reaction yields a substantial specific activity, making it suitable for RSV applications. Rhenium-186, a non–bone-seeking radioisotope, is an excellent choice for RSV due to its nonresidualizing properties and lack of prolonged retention in the body or lymph nodes, even in cases of extra-articular leakage.

In Europe, commercially available ^186^Re-labeled particles are used for RSV. The recommended activity levels for RSV with [^186^Re]Re-sulfide colloid are specified for different joints. For the hip and shoulder joints, the range is 74–185 MBq; for the elbow joint, it is 74–111 MBq; for the wrist joint, it is 37–74 MBq; for the ankle joint, it is 74 MBq; and for the subtalar joint, the range is 37–74 MBq. When treating multiple joints in one session, the total activity administered should not surpass 370 MBq [252–257].

The utilization of ^186^Re and the complexities in preparing radiolabeled particles for RSV have spurred fascinating research and innovative approaches [104, 154, 184, 249–257]. Like other radionuclides, ^186^Re presents a range of advantages, disadvantages, and practical applications. The potential use of ^186^Re in RSV holds great promise due to its efficient production logistics, optimal tissue penetration depth, and γ emission suitable for external imaging with a standard gamma camera. However, the need for enriched ^185^Re target for ^186^Re production leads to reduced cost-effectiveness compared to ^166^Ho in RSV applications.

#### 4.6.8. Rhenium-188

Rhenium-188 decays to ^188^Os with a half-life of 16.9 h, emitting β^−^ particles with a maximum energy of 2.11 MeV and a tissue penetration depth of 3.5 mm. Moreover, it releases γ photons of 155 keV (15%). The β^−^ emission from ^188^Re has sufficient energy to penetrate the thickened synovial membrane, allowing treatment of larger joints like the knee. The predominant 155 keV γ emissions enable scintigraphic imaging for therapeutic monitoring, leak detection, and dosage calculation. The half-life of ^188^Re is suitable for preparing radiolabeled particles, QC studies, and safe therapeutic effects without residual harm. In comparison with ^186^Re, ^188^Re is preferred for RSV applications due to its availability in a NCA form from a ^188^W/^188^Re radionuclide generator in hospital radiopharmacies [258]. The long half-life of the parent radionuclide, ^188^W, ensures generator functionality for several months. Rhenium's versatile chemistry, featuring eight potential oxidation states, enables its binding to a wide range of microparticles with diverse properties, enhancing its adaptability for various medical applications [259]. The tissue penetration depth of ^188^Re is equal to that of ^90^Y, but its shorter half-life reduces radiation dose and minimizes leakage to nontarget organs.

The appealing aspects of utilizing ^188^Re in RSV for large joint treatment include its ideal half-life, energetic β^−^ particles, detectable γ photons, and on-demand availability from a ^188^W/^188^Re generator. However, the primary challenge in the widespread use of ^188^Re-based RSV agents is the cost-effective access to the ^188^W/^188^Re generator. The extensive use of ^188^Re in RSV treatment highlights the significant clinical value of this radionuclide [152, 182, 260–275].

#### 4.6.9. Lutetium-177

Lutetium-177, with a half-life of 6.7 d, undergoes decay to yield stable Hafnium-177 (^177^Hf). In most cases, ^177^Lu transitions to the ground state of ^177^Hf, emitting β^−^ particles with a maximum energy of 0.497 MeV. Alternatively, in a smaller percentage of instances, it decays to excited states of ^177^Hf, emitting β^−^ particles with energies of 0.384 and 0.176, 0.250, and 0.321 MeV above the ground state before returning to the ground state through photon emission. Throughout the decay process, ^177^Lu emits low-energy γ photons at 113 and 208 keV, which help in scintigraphic imaging applications [276, 277].

Lutetium-177 can be produced through two different routes, namely, direct neutron activation, which involves irradiating a target of natural or enriched (in ^176^Lu) lutetium oxide (Lu_2_O_3_) and indirect production, where enriched (in ^176^ Yb) ytterbium oxide (Yb_2_O_3_) is irradiated, followed by radiochemical separation of ^177^Lu from ytterbium isotopes. These two methods result in products with different specific activities and radionuclidic purities. Additionally, the cost of ^177^Lu obtained from these methods varies significantly. Direct neutron activation of natural Lu_2_O_3_ is particularly advantageous due to the high thermal neutron capture cross-section of ^176^Lu (σ_ther__mal_ = 2100 b). The specific activity achieved through this method is sufficient for the preparation of ^177^Lu-based agents for RSV application. To ensure the RSV procedure remains cost-effective and accessible to a larger population, it is preferable to use carrier-added ^177^Lu, which is relatively more affordable while still meeting the necessary therapeutic requirements. Detailed discussions on ^177^Lu production methods are available in the contemporary literature sources [278–282].

Lutetium-177 emits β^−^ particles with a mean range of 670 μm, making it highly suitable for RSV procedures. This penetration depth is optimal for targeting the synovial tissue while minimizing damage to surrounding healthy structures, enhancing the safety and effectiveness of RSV treatments. The low-energy γ photon emission supports simultaneous scintigraphic imaging for joint evaluation, leakage analysis, and dosimetry purposes. Lutetium solely exists in the +3-oxidation state, simplifying its solution chemistry without redox complications. The chemistry of ^177^Lu is adaptable for conjugation with various particles using standard radiolabeling techniques. The longer half-life of ^177^Lu presents logistical advantages for transportation to distant nuclear medicine centers. However, the possibility of radiation exposure due to the accidental leakage of the instilled particles owing to its long half-life can be a drawback of using ^177^Lu-based agents for RSV application.

The tremendous prospects associated with the use of ^177^Lu have culminated in the development of numerous ^177^Lu-based radiopharmaceuticals for RSV, which have been scrupulously evaluated for their efficacy, commencing with preliminary physicochemical suitability followed by in vitro and in vivo studies using animal models, and ultimately progressing to clinical settings. In recent decades, extensive research has been dedicated to developing different therapeutic agents utilizing ^177^Lu [173, 174, 179, 283–289]. A variety of particles endowed with diverse chemical attributes have been utilized for radiolabeling, including tin colloid [289–292], chitosan [140], zirconia colloid [284], phytate complex [288], and hydroxyapatite [173, 174, 293]. Notably, tin colloid and hydroxyapatite have garnered the most attention and have been employed in the treatment of various joints, ranging from small to large, with notable therapeutic success.

#### 4.6.10. Yttrium-90

With a half-life of 64.1 h, ^90^Y transforms the stable Zirconium-90 (^90^Zr) by emitting high-energy β^−^ radiation (maximum energy of 2.28 MeV and mean energy of 0.935 MeV). The β^−^ radiation releases around 80% of its energy within the initial 4–5 mm, making it an ideal radionuclide for intra-articular RSV of the knee joint and patients with thickened synovial tissue. The 64.1-h half-life proves to be convenient for radiolabeling, performing QC tests, and administering the preparation to patients. Throughout the treatment process, the ionization remains consistent enough to eliminate synovial tissue and reduce synovial fluid production. The ^90^Sr/^90^Y generator ensures immediate availability of ^90^Y in NCA form either at the hospital or at a centralized radiopharmacy [294]. Additionally, ^90^Y with sufficient specific activity for RSV procedure can be produced through direct neutron activation of natural yttrium target in a moderate-flux research reactor. Yttrium primarily exists in a tri-cationic state and can be easily combined with various particles using standard radiolabeling techniques.

The appeal of ^90^Y in RSV primarily stems from its favorable nuclear decay properties, excellent chemical characteristics, and ease of availability through a radionuclide generator system. Despite its remarkable nuclear and chemical properties, ^90^Y does have certain limitations. The lack of γ photon emission from ^90^Y poses a difficulty in traditional scintigraphic imaging and assessing the distribution of radioactivity post-therapy. Any possible leakage from the joint could result in significant radiation doses to different organs such as the lymph nodes, liver, and kidneys.

Unquestionably, ^90^Y emerges as the most commonly used radioisotope for treating hypertrophic and exudative knee synovitis in individuals dealing with various rheumatic diseases, including RA, osteoarthrosis, and peripheral spondyloarthropathies (psoriatic arthritis and ankylosing spondylitis) [32, 37, 105, 142, 143, 147–151, 178, 183, 295–322].

The characteristics, advantages, and limitations of therapeutic radionuclides used in RSV are summarized in Table 1.

## 5. Limitations of RSV

Although RSV is a minimally invasive procedure with multiple advantages, it also has several limitations:

• RSV is a sophisticated medical procedure that requires a multidisciplinary approach [38]. Its success heavily depends on the availability of a hospital nuclear pharmacy and a specialized team that includes a rheumatologist, orthopedic specialists, radiologist, nuclear medicine physician, clinical hematologist, and physiotherapist.
• RSV is not a universal treatment for all inflamed joints and its applicability depends on the specific joint pathologies of each patient [323]. A thorough understanding of the pathophysiology of synoviopathy is crucial, as the treatment's efficacy is influenced by factors such as the severity of synovitis, the intensity of the inflammatory response, the stage of arthrosis, and the overall level of systemic inflammation.
• The response to RSV is not immediate but follows a lag phase that can extend for several weeks to months. Larger joints typically respond more quickly than smaller ones, with notable improvement in the knee joint observed within 4 to 6 weeks, whereas interphalangeal joints may take up to 4 to 6 months [38].
• If there is no significant response, additional injections are not recommended after two failed treatments, ensuring a minimum interval of 6 months between the administrations. Failure to achieve efficacy after two consecutive RSV procedures serves as a contraindication for further radionuclide- based therapies [324].
• Ensuring the success of the treatment requires the availability of the necessary radioisotope in the required quantity at the time of therapy, posing an additional challenge.
• Although RSV is an outpatient procedure that does not require hospitalization, post-treatment mismanagement and challenges in patient self-care have emerged as significant obstacles [325].
• Strict radiation safety regulations require the availability of specialized facilities and trained personnel.
• Despite its numerous advantages, RSV remains underutilized due to limited accessibility.

## 6. Regulatory Issues of Radiopharmaceuticals Used in RSV for Clinical Use

No conversation concerning RSV can be considered comprehensive without addressing the intricate realm of regulatory matters. The regulatory framework stands as the pivotal element influencing the practical application of novel RSV agents, consequently, fostering innovation. While the development of new radiolabeled microparticles for RSV is crucial, their advancement and clinical application may be hindered by complex legal and regulatory challenges. Even though there are positive advancements, there is still a long road to travel before new radiolabeled microparticles can be easily incorporated into RSV for regular clinical use. Success in this endeavor depends on excellence at every stage of development, from preclinical research to regulatory approval and market accessibility.

The utilization of radionuclides in RSV is classified as an active pharmaceutical ingredient (API) due to its role as a foundational component for making radiolabeled microparticles intended for human use. Consequently, the deployment of radionuclides and radiolabeled microparticles in RSV falls under the purview of numerous national and international guidelines, regulations, and statutes. Beyond adhering to radionuclide specifications, the production protocols for radiolabeled microparticles must strictly conform to the tenets of current good manufacturing practice (cGMP), which delineates the fundamental criteria for clinical utilization. The enforcement of cGMP aims to safeguard patients from potential risks arising from inadequate safety and quality standards, while also fostering uniformity in the application of regulatory protocols. Compliance with regulations requires meticulous planning of the production process and the establishment of a robust quality management system. This includes adherence to cGMP standards, QA, QC, lifecycle management, and risk mitigation measures. Additionally, it is essential to meet the quality and purity standards set for radiolabeled microparticles.

The guidelines put in place by regulatory bodies for the production and acceptance of radiolabeled microparticles must be meticulously recorded in a Drug Master File (DMF). Approved rules outline the manufacturing of radionuclides used as an API following cGMP, as specified in the Code of Federal Regulations. Key elements of cGMP include the exact definition and careful management of production procedures for radiolabeled microparticles, ensuring uniformity and compliance with approved standards. A dedicated department should oversee QA and QC, either through separate units or a single team or individual, depending on the organization's size and structure. Importantly, critical phases of the formulation processes for radiolabeled microparticles and significant changes in processes must undergo comprehensive validation. Adequate staffing of well-trained personnel, suitable facilities, proper equipment, approved procedures, and secure storage and transportation amenities are also imperative for maintaining operational efficiency.

When engaged in the production of radiolabeled microparticles, it is essential to adhere to the requirements outlined in the “Guidelines on Good Radiopharmacy Practice” and “Guidance on Current Good Radiopharmacy Practice (cGRPP).” Submission of a dossier for each batch of radiolabeled microparticles before distribution is a prerequisite. Proper measures must be implemented to ensure the storage and handling of radiolabeled microparticles in order to maintain the necessary quality throughout their shelf-life. The analysis of radionuclides used in radiolabeling and microparticles should adhere to pharmacopeia standards and regulatory requirements, with the exception of sterility and pyrogen tests, which are performed on batch samples.

Manufacturers involved in the consistent production of radiolabeled microparticles must satisfy pharmaceutical GMP regulations and typically hold a license from a Nuclear Regulatory Authority (NRA). Manufacturers are required to show that the facility can protect health and reduce the chances of harm to people or property. Compliance with radioactive material handling, radiation protection programs, and management procedures is also required. When transporting radiolabeled microparticles, different regulations apply compared to regular pharmaceutical components. National and international authorities have established specific guidelines for the safe transportation of radioactive materials from the manufacturer to the end user.

## 7. Conclusion and Future Outlook

This review presents a thorough perspective on RSV, spanning from basic exploration to practical application across a wide range of platforms. The extensive array of references highlighted in this review indicates the rapid evolution of RSV, with ongoing exploration and the emergence of promising radiolabeled microparticles. These advancements are set to enable the development of innovative radiolabeled microparticles with unique properties, catering to both current and future medical needs. Radiopharmaceutical scientists must evaluate different strategies to ensure a stable and dependable supply of radiolabeled microparticles for immediate and long-term demands in the realm of RSV.

The success of RSV depends not only on constant improvements in using new radionuclides, but also on choosing various types of microparticles designed for different joints to achieve positive therapeutic results. The implementation of RSV with new radionuclides and microparticles also demands expertise in radiolabeling techniques. Despite the rapid advancements in treatment methods and the fast pace of innovation offering promising options, successfully integrating these breakthroughs into clinical practice for RSV requires a blend of expertise, adaptability, and artistic creativity. The essence of this artistic skill lies in the ability to produce next-generation radiolabeled microparticles for RSV that can meet the growing therapeutic needs.

Although the advancement of RSV through emerging radionuclides and microparticles shows great potential, securing regulatory approval for radiolabeled microparticles is essential for clinical application. The emphasis on the quality of radiolabeled microparticles is particularly evident, as not only must the radionuclide adhere to stringent standards, but the radiolabeled microparticle must also satisfy established regulatory requirements. Any new radiolabeled microparticles must be tailored to align with local laws, regulations, and institutional guidelines.

The current method of utilizing radiopharmaceuticals for RSV in nuclear medicine facilities is poised to shift, with future distribution likely to be handled by centralized radiopharmacies established to meet cGMP standards. Ensuring cGMP compliance for radiopharmaceuticals intended for RSV involves a strong emphasis on product quality, operational systems, validation, and production documentation. The accuracy and completeness of production records are highly valued by regulatory bodies. The trend in radiopharmaceuticals for RSV is shifting toward user-centric design, prioritizing simplicity, and ease of use, rather than requiring users to adapt to complex procedures.

Automating the production process is a straightforward approach to meeting GMP standards while ensuring consistent product quality. It enhances productivity, maintains quality uniformity, minimizes radiation exposure for employees, reduces human errors, and enables comprehensive record-keeping of all activities. This automation also allows for the comprehensive collection of production records in line with cGMP standards. Despite the numerous benefits offered by automated systems, there are still specific unplanned manual tasks that need to be done. Accelerating the integration of automation in production technology is crucial to lay a strong foundation for future progressions.

In conclusion, there is growing interest in the use of value-added radiolabeled microparticles for RSV. While efforts have been made to accelerate product development, a gap remains between laboratory research and clinical application, despite academic initiatives fostering collaboration. Bridging this gap requires coordinated efforts among scientists, clinicians, hospitals, industry leaders, and regulatory authorities to overcome challenges related to production technology and compliance. Continued commitment from all stakeholders is crucial to securing a promising future for RSV in nuclear medicine, building on its past successes and current advancements.

## Figures and Tables

**Table 1:** Characteristics, advantages, and limitations of therapeutic radionuclides used in radiation synovectomy.

Radionuclide	Half-life	Max. β^−^ energy (MeV)	γ-energy (keV)	Advantages/limitations
^198^Au	2.7 d	0.96	412	Easy production and simple radiochemical processing, adequate half-life for formulation and supply of radiopharmaceuticals Presence of high-energy γ component, significant radiation exposure in case of leakage of particles

^165^Dy	2.33 h	1.29	94	Short half-life mitigates the impact of possible leakage, presence of suitable energy γ photons in low abundance helps in imaging, straightforward production and simple radiochemical processing Short half-life presents logistical problem

^169^Er	9.4 d	0.35	Negligible	Suitable β^−^ energy for treating digital joints, simple reactor-based production, long half-life helps in efficient distribution of radiopharmaceuticals Requirement of enriched target, negligible proportion of the γ component making post-therapeutic scintigraphy challenging

^32^P	14.3 d	1.71	—	Availability in NCA form at a reasonable cost, relatively long half-life helps in easy distribution of radiopharmaceuticals Lack of detectable γ photons making post-therapeutic scintigraphy challenging, very high radiation dose to both articular cartilage and bone in case of leakage of particles

^166^Ho	26.83 h	1.85	81	Comparatively shorter half-life mitigates the risks associated with the possible leakage of the particles, presence of low-abundance imageable γ photons helps in scintigraphy, cost-effective production Short half-life brings logistical challenges

^153^Sm	46.27 h	0.81	103	Presence of suitable energy γ radiation helps in imaging, favorable chemistry for radiolabeling, easy production, and simple radiochemical processing Logistical challenges owing to not-so-long half-life, relatively high γ abundance (28%) poses a risk of collateral damage, requirement of enriched target increases the production cost

^186^Re	90 h	1.08	137	Long half-life provides logistical advantages, presence of imageable γ photons helps in post-therapy scintigraphy, easy production in nuclear reactor Requirement of enriched ^185^Re target, complex chemistry of Re in comparison with that of trivalent lanthanides

^188^Re	16.9 h	2.11	155	Availability on-demand in NCA form from ^188^W/^188^Re generator, presence of suitable energy γ protons for post-therapy imaging Intricate chemistry compared to that of trivalent lanthanides, availability, and cost of ^188^W/^188^Re generator

^177^Lu	6.7 d	0.49	208	Relatively longer half-life helps in the distribution of radiopharmaceuticals, possibility of post-therapy imaging due to the presence of low-energy γ photons, favorable chemistry, easy production Possibility of radiation exposure in case of leakage of particles

^90^Y	64.1 h	2.28	—	Relatively longer half-life provides logistical advantages, availability on-demand in NCA form from ^90^Sr/^90^Y generator, favorable chemistry Absence of γ photons, significant radiation doses in case of leakage of the particles

## Data Availability

All data related to this study are included within the manuscript.
